# The experience of provided information and care during pregnancy and postpartum when diagnosed with preeclampsia: A qualitative study

**DOI:** 10.18332/ejm/139488

**Published:** 2021-09-08

**Authors:** Maria E. Andersson, Christine Rubertsson, Stefan R. Hansson

**Affiliations:** 1 Obstetrics and Gynecology Unit, Section V, Department of Clinical Sciences Lund, Faculty of Medicine, Lund University, Lund, Sweden; 2 Skåne University Hospital (SUS), Lund, Sweden; 3 Department of Health Sciences, Faculty of Medicine, Lund University, Lund, Sweden

**Keywords:** complicated pregnancy, experience, information, obstetric care, qualitative methods, preeclampsia

## Abstract

**INTRODUCTION:**

Despite preeclampsia being one of the most severe obstetrical complications there is only scant research describing women’s experiences of preeclampsia. The aim of this study was to explore women’s experience during pregnancy and the postpartum period regarding the provided information and care concerning preeclampsia.

**METHODS:**

A qualitative study was designed. Semi-structured face-to-face interviews were performed with fifteen women who were diagnosed with preeclampsia and included at two maternity units located in southern Sweden. The material was analyzed using content analysis.

**RESULTS:**

Suffering from preeclampsia was understood as being stressful, illustrated in four themes: fragmented information, lack of care planning, separation postpartum, and overall stress and worry.

**CONCLUSIONS:**

The women experienced fragmented obstetrical care and information deficits when diagnosed with preeclampsia. Our findings indicate a need for additional support and professional guidance due to increased stress, worry, and despair of being separated from the newborn. Future research investigating specific care-planning and postpartum follow-up are suggested as steps to improve care for women with a pregnancy complicated by preeclampsia.

## INTRODUCTION

Preeclampsia (PE) is one of the leading causes of both fetal and maternal morbidity and mortality. Yearly, around 3–7% of all pregnancies are affected, corresponding to a total of 8.5 million women worldwide^1^, of which 5000 occur in Sweden^2^. According to the National Swedish statistical database registry for pregnancy, the incidence of PE was 3.0% in 2019^3^. Preeclampsia is characterized by gestational hypertension together with proteinuria and/or other organ manifestations and/or fetal growth restriction, arising after 20 weeks of gestation^2,4^. To date, there is only symptomatic treatment available, the only ‘cure’ is to give birth either by induction of labor or by Caesarean section.

Preeclampsia is considered a syndrome and the etiology is still not fully understood. There are several known risk factors for PE, such as autoimmune diseases, diabetes, renal disease, chronic hypertension, own or familial history of PE, body mass index (BMI) >30 (kg/m^2^) and ethnicity, particularly women of African descent have an increased risk^2,5^. Women with hypertensive disorders of pregnancy are also at an increased risk of developing postpartum depression, anxiety, and post-traumatic stress disorder^6^. Adding to this, it has been shown that women and their babies affected by PE, have an increased long-term risk of developing hypertension, stroke, and cardiovascular disease later in life^1,7^.

The Swedish Maternal Health Care system (MHC) provides screening for all pregnant women during the antenatal care program, with the midwife as the primary caregiver^8^. During a normal pregnancy, 6–9 visits to the midwife are recommended, but does not include routine visits to a doctor. According to the competence description for Swedish midwives, the midwife possesses the competence to: ensure patient-safe, person-centered, equal, available, and continuous care to adapt the care provided according to the individual needs of the patient^8^. Information and support must be provided so that the women can make their own decisions^9^. In fact, the MHC was introduced in 1950s with the main purpose to identify PE at an early stage. Screening involves blood tests, urine samples for protein analysis and blood pressure measurements^2^. For women, and their relatives, information is available at a public internet-based care guide: www.1177.se (The National Healthcare Guide 1177 services, available in Swedish and translated to different languages), providing information about the pregnancy and about PE^10^. When high blood pressure is identified, the women are referred to a hospital-based specialized obstetric care unit. To date, antenatal care is the most important contributing factor to healthcare to reduce maternal mortality in modern times^8^.

Despite PE being one of the most severe obstetrical complications, there is only scant research reports describing women’s experiences of PE and care. In the few qualitative studies that have been published, women often describe PE as a frightening and life-threatening condition, with lack of care and low psychosocial support, experiences that often collide with their expectations of pregnancy and childbirth^11,12^. Furthermore, there is a lack of knowledge regarding the understanding about the woman’s and family’s needs^13,14^, the need of support during hospital stay^12^ and access to information^14^. These studies highlight an unmet need for information and understanding about PE.

The aim of the study was therefore to explore women’s experiences during pregnancy and the postpartum period regarding the provided information and care concerning PE.

## METHOD

### Setting

The study was performed at two maternity units in southern Sweden. The units had a total of 9012 births in 2019 and the incidence of PE was 3.4%, with 0.3% as early onset (<34th week) PE^3^.

### Design

A qualitative descriptive research design with an interview guide was used (Table 1) and semi-structured face-to-face interviews were performed. The interview guide was designed based on clinical experience and confirmed after one pilot interview that resulted in no changes^15,16^. The questions were open-ended, allowing women to speak openly about the provided information and care concerning PE during pregnancy, at the time of diagnosis and postpartum. The narratives were followed up with subsequent questions, to gain a deeper understanding of the women’s experiences such as: ‘When did you realize you had preeclampsia and if you have any suggestions about your care, what kind of change would that be?’.

**Table 1:** Interview guide

***Engagement question***
Can you tell us what information you received at the maternal healthcare, when you were told that you had preeclampsia and what information you received during the rest of your pregnancy and before you left the hospital?
***Subsequent questions***
When did you realize you had preeclampsia?
What did you know about preeclampsia before pregnancy?
How did you get the information and how did you experience the way to get the information?
What challenges were experienced in connection with information? When and how often would you like it?
What were your information needs and how were they met?
Can you describe if something felt difficult when you got the information?
If you had to change something, what would you change?
***Exit question***
Is there anything additional you would like to say about the information and the care you got about preeclampsia?
***Probes in order to minimize misunderstandings***
Can you please say more about this?
Can you give an example of that?
Can you tell me something else about that?

### Data collection

Inclusion criteria were primiparous women diagnosed with PE and multiparous women with ongoing PE without a history of PE in a previous pregnancy. All the women were fluent in Swedish, but one preferred to speak in English during the interview. The participation was based on voluntary decisions and informed consent was signed before the interview. The participants were recruited consecutively between July and December 2019. The first author (MA) visited or called the hospitals daily, to identify possible patients to be included. The women were asked about participation at the maternal healthcare unit or in the neonatal intensive care unit (NICU) or by telephone if discharged from hospital. Two women declined to participate in the study. The interviews were performed 1–6 weeks postpartum at a mutually agreeable place such as the women’s private home or a separate room at the hospital. The interviews were conducted until no additional information was obtained from the participating women, when saturation was reached^17^. The study was approved by the Regional Ethics Board of Lund University (2019–04240).

### Data analysis

The analyses were conducted in two steps, the first step, according to a manifest analysis and the second step as a latent content analysis, with an inductive approach, based on Graneheim and Lundman^18^. All the interviews were audio-recorded and transcribed verbatim by the first author^16^. The interviews lasted on average 25–50 min. To initiate the data analysis, the interviews were listened to again and then read through several times to gain an overall assessment and understanding, always with the study’s purpose in mind. From the transcripts, patterns were identified followed by extracting words and sentences that were relevant to the study assigned as ‘meaning units. These meaning units were then condensed, while keeping the core of the text and assigned to certain codes^18^. The codes were compared and grouped by two independent investigators (MA, CR) into sub-themes on the basis of their similarity. The sub-themes were further aggregated into themes based on the underlying meaning expressed in the sub-themes. The various codes were compared based on differences and similarities and further sub-themes, which constituted the manifest content. Examples of the analysis process from meaning units to themes are given in Table 2. Finally, the underlying meanings, that is the latent content of the sub-themes, were formulated into four themes given in Table 3. All authors checked the final analysis for integrity and participated in the final interpretation of the data.

**Table 2:** Examples of the analysis process from meaning units to themes

*Meaning unit*	*Condensed meaning unit*	*Code*	*Sub-theme*	*Theme*
At the maternity ward itself … there was not much information	Did not get much information on maternity care	Not so much information on maternity care	Missing overall information	Fragmented information
It feels like no one was talking to me … about it … risks … the pros and cons of getting induced … so I could prepare somehow	No one talked about the risks, pros and cons of induction, so you could prepare	No information about risks, pros and cons to be able to prepare	Inconsistent information	Fragmented information
A seated conversation with a doctor at the time of discharge … a longer conversation afterwards … and not only that the samples were good and here you get a note	Seated conversations with doctors at discharge, longer conversations about test results and more	Seated explanatory conversation with the doctor at the time of discharge	Individualized plan for treatment and follow-up	Lack of care planning
There they would probably have wanted … on BB the child and mother are together … and there they have control of both	Wish that mother and child are together as at BB and have control of both at the same time	The desire for mother and child to be cared for together	Despair of being separated from the newborn	Separated postpartum
It felt like you were left … while I was still in the hospital … here they only cared about him … and they pushed me to have him in my arms for several hours … and I felt bad … it became a small collision between neonatal and my preeclampsia … I felt a little neglected	They cared of only the child and that he would be in my arms even though I felt bad. Felt neglected.	A feeling of being alone and neglected	A feeling of coming in second place	Separated postpartum

BB: maternity hospital in Sweden.

**Table 3:** Four themes and ten sub-themes that emerged from the data

*Themes*	*Sub-themes*
**Fragmented information**	Missing overall information Lack of knowledge and understanding of health risks Inconsistent information Difficulties to ‘take in’ information
**Lack of care planning**	Individualized plan for treatment and follow-up Timing and involvement in care planning
**Separated postpartum**	A feeling of coming in second place A despair of being separated from the newborn
**Overall stress and worry**	Experiencing stress and worry A request for both oral and written information

## RESULTS

In total, fifteen women diagnosed with PE were included, of which six developed severe PE and two the HELLP syndrome. Their characteristics are described in Table 4. The participating women’s experiences were illustrated in four themes: 1) Fragmented information, 2) Lack of care planning, 3) Separation postpartum, and 4) Overall stress and worry; with ten sub-themes as presented in Table 3, and based on quotations from the interviews.

**Table 4:** Characteristics of women with preeclampsia in the study (N=15)

*Characteristics*	*n (%)*
**Age** (years), mean ± SD	30.8 ± 7.11
**BMI**, mean ± SD	25.2 ± 4.6
**Primigravity**	14 (93)
**Medical condition^a^**	
High blood pressure	1 (6)
Family high blood pressure	4 (27)
Diabetes	1 (6)
In vitro fertilization (IVF)	2 (13)
Lichen planus	1 (6)
**Gestational age**	
Preterm birth*	9 (60)
Term birth**	6 (40)
**Mode of delivery**	
Vaginal	8 (53)
Induction	6 (40)
Assisted/vacuum extraction	2 (13)
Caesarean section	7 (47)
**Newborn in NICU**	6 (40)
**Days in hospital care**	
2–6	7 (47)
7–11	5 (33)
12–19	3 (20)

* Birth before 37 weeks of pregnancy (27+3 – 36+6).

** Birth after 37 weeks of pregnancy (37+4 – 40+3).

a Some women had more than one medical condition.

BMI: body mass index (kg/m^2^).

### Fragmented information

The women described as insufficient as well as inconsistent information about PE throughout the whole period: during pregnancy at the MHC, the hospital stay, and the postpartum period. In particular, the information given by several health professionals was fragmented and did not inform about the long-term health consequences. Most importantly, women with PE also described difficulties to ‘take in’ information during this stressful period and pointed out that the information was given en passant, not focused on a designated time such as the ward rounds.

*Missing overall information at the MHC*

The women described that in general they received a lot antenatal information, but the main focus was on a normal pregnancy. Many women were not able to explain why they had been monitored with blood pressure and tested for proteinuria. The midwife told them about the screening results, but they received little or no specific information about PE:

*‘Now (postpartum) I've understood why they take a blood pressure every time one is there. I didn't realize why before.’* (PE15)

All women with high blood pressure received information about what further PE symptoms to be aware of. However, they were not given any detailed explanation about PE, just the fact that it could become a serious pregnancy complication.

*Missing overall information during the hospital stay*

When the women were admitted to the hospital, due to risk for or confirmed PE, they described that they received inadequate information. They had to stay at the hospital for further observation but did not get ‘the whole picture’ or information about the specific screening procedures, including Doppler ultrasound measurements and the extended blood analysis. Some women did not understand their situation until the postpartum period:

*‘… it feels like for the first time I actually got some kind of adequate information… it was after … the birth … and that feels a bit strange … I spent almost two weeks in the hospital.’* (PE9)

*Missing overall information postpartum*

Even when discharged from hospital, many women reported that the given information about PE was too brief and was missing details about when and whom to visit for a check-up. No information about further risk of developing PE in a subsequent pregnancy or on the long-term health consequences, such as cardiovascular diseases:

*‘… it is certainly something that they could have mentioned … that until next time … that there's a greater likelihood I will get it the next time.’* (PE4)

The two women who developed the HELLP syndrome also experienced poor continuity and information postpartum. They received no information during the ward rounds, just secondhand information given by the nurses or midwives. Leading to some advice from one woman:

*‘ … at the maternity ward … one of the first days I was there … a doctor should have come and talked to me … “this is how the caesarean section went and this is how the situation looks now”… not wait until the day of discharge … I knew they had rounds … but the rounds never came to us.’* (PE8)

*Understanding of future health risks*

Overall, most women reported poor knowledge about PE and its long-term consequences. Many reported little understanding of their own health risks and did not know that PE could progress into eclampsia, especially that it could also continue into the postpartum period. The women who developed early-onset PE and had no high blood pressure at the MHC, had no knowledge about PE and, that symptoms, including headaches and signs of sickness, could be indicative of more severe PE. It also emerged that their partners and relatives had little knowledge about PE. Some women also expressed that they had little knowledge to be able to ask the right questions. They did not know what to ask and they were often left with several unanswered questions:

*‘… that they explain even more … even if they ask me … “have you understood”… but I don't know what to ask … because I don't even know what they are talking about.’* (PE10)

Few women received information about future health risks or could describe what they should be aware of in a future pregnancy.

*Inconsistent information*

Several women reported that they received inconsistent information, often given in unknown medical language. Furthermore, doctors gave slightly different information. The women felt powerless that they had minimal or no involvement in the decision making concerning them:

*‘… nowhere in this whole process have I been involved about … that I haven't been given a diagnosis… the decision to be hospitalized … the decision to be induced (for labor) … nobody has explained the chain of events to me … [crying]… I had to accept all the decisions.’* (PE14)

*Difficulties to ‘take in’ information*

Several women did not realize the severity of the illness, until the time of discharge from the hospital or, in some cases, first several weeks postpartum. Some describe that they experienced a feeling of not been able to ‘take in’ the given information during the acute phase. However, when they were feeling better, they expressed a need for more information:

*‘I got some information there (at hospital) … that I didn't really take in … it was a little hard to grasp and understand.’* (PE11)

### Lack of care planning

Although the interview guide focuses on experiences about the given information on PE, the women expressed an urgent need for involvement in their own care and for care planning during the whole process, when admitted to the hospital, during the hospital stay, and in the postpartum period.

*Individualized plan for treatment and follow-up*

Overall, women described that no care planning involving them was done throughout their hospital stay. No caregiver took time to sit down and explain the different steps in the management plan, what choices and medical alternatives were available, and their potential outcomes:

*‘… that no doctor came afterwards and sat down and talked … about the c-section at all and what happens … and what I can expect now …’* (PE8)

*Timing and involvement in care planning*

The lack of care planning and information led to the fact that women did not understand the seriousness of the possible consequences of PE. They requested repeated information at a more appropriate time point when they felt better and when the situation regarding their baby was more stable. Some asked also for a consistent and summarized information regarding their situation:

*‘It would have been nice (to understand) what is connected … why you go to a growth assessment, ultrasound … why you go to a blood flow measurement … how all these parts come together … how that can affect me and how it can affect my child.’* (PE3)

### Separation postpartum

When the newborn was admitted to NICU several hurdles challenged their situation.

*The feeling of coming in second place*

Postpartum, the women described their despair and feelings of being abandoned. They were tired, had pain and tried to overcome the symptoms of PE. They felt sick and, at the same time, they wanted to be close to their baby; the neonatal staff also pointed out the importance and impact of skin-to-skin care. In addition, they were adjusting to motherhood. The need for support and encouragement from all the healthcare professionals was obvious in order to overcome the feelings of being alone in a difficult situation. The women separated from their children at NICU requested additional support and care:

*‘It felt like you were left alone … at the same time as I was still in the hospital … here they only cared about him … and they were pushing for him to be in my arms for several hours … and I was feeling ill … it became a little clash between the neonatal unit and my preeclampsia … I felt a little pushed aside.’* (PE10)

*A despair of being separated from the newborn*

In addition, the women expressed stress when separated from their newborn and that they were too far away from their newborn at the NICU. The women also expressed the view that the staff should have the competence to be able to take care of both the mother and child as in the maternity ward in order to avoid separation:

*‘That when you're prematurely born, should be the same as it is on the maternity ward … because there … babies and mothers are together… and there they check up on both.’* (PE3)

### Overall stress and worry

The overall lack of information, lack of care-planning, and concerns about future health risks, made the women stressed and worried, underlining the need for both oral and written information. Instead, or as a complement to missing information, they had to search on the internet.

*Experiencing stress and worry*

Many women also described concerns about their unborn child and were, in general, less concerned about themselves. They described specific stressful situations such as when their blood pressure was high or when the blood analysis was abnormal:

*‘… you are scared “shitless”… that you're going to … that your body is going to shut down.’* (PE10)

Several women expressed that the word ‘toxemia’ PE itself, is signaling danger, building up even more stress and worries. At the same time, they expressed that the missing information in itself was frightening. They wanted information about the worst-case scenarios, even if it scared them:

*‘There was very little information about … what it can lead to … and maybe you don't want to say that … because you don't want to scare someone.’* (PE11)

Several of the women in the study were emotionally affected by their experiences. Some cried during the interview and others were angry over their situation. They also described their concerns about a future pregnancy and the fear of becoming pregnant again.

*A request for both oral and written information*

Many of the women expressed a wish to receive both oral and written information. They wanted written information to be able to read when the chaos had settled down and not feel so stressed:

*‘I would have liked more written information … about what preeclampsia is … and what could happen … so that you … can re-read it in peace.’* (PE8)

In order for the women to better understand their situation, they either asked friends or relatives or searched for information regarding symptoms, treatments and risk factors on the internet. The women searched for specific information, however, the results made them more confused and worried:

*‘I have made sure to read up on it myself too … on the National Health Information website … read on the internet … to fill in the blanks a little bit … um … but it's basically the same information … the symptoms.’* (PE11)

## DISCUSSION

This study explored women’s experiences during pregnancy and the postpartum period regarding the provided information and care concerning PE. The interview guide focused on the experiences of the given information about PE, but the women reported an urgent need for involvement in their care based on individual needs. The findings suggest that experiencing PE involved strong feelings of uncertainty and stress, described as: 1) general lack of information during antenatal and postpartum care; 2) a need for more support, due to increased stress and worry; 3) a despair of being separated from the newborn; and 4) a profound lack of knowledge regarding PE and the associated long-term health risks.

The women experienced a general lack of information, and which was fragmented. Repeated consistent information in a person-centered way could be more effective and helpful in meeting their needs, as revealed in our interviews. In addition, the data indicate that many women had difficulties in understanding the information they received regarding their condition and why they had to stay in the hospital. Healthcare professionals must ensure that women in this situation receive the information they need and that the information is understood^19^. Appropriate communication is one of the most difficult tasks in healthcare and several of the women did not experience involvement or informed consent in the medical decisions. Women in this study felt that they received information without understanding the options or the advantages and disadvantages with the planned interventions. A person-centered approach and continuity of care is suggested to be more beneficial, which has been shown as a successful strategy for a mixed-risk population in a review by Sandall^20^. The participating women also felt that they had difficulties to ‘take in’ the information. One explanation could be that PE causes general swelling of the central nervous system^21^. Other studies have suggested an association between PE and cognitive functions^22^, further supporting the idea that it may be difficult to understand given information in the acute phase of the disease. In addition, many of the women with severe PE, who were separated from their newborn, particularly expressed stress, which is another factor that may influence the understanding of given information. There are also studies showing association between PE/HELLP and depression^23^ and should be considered in future research.

As a consequence of the fragmented and limited information, many of the women felt extra stress and worry. Most of them expressed a concern for their unborn child when they were sick or got the diagnosis, rather than thinking about themselves. Similar findings have been described in an Australian study, where the majority (84.6%) of women reported that they were more worried about their babies^14^. The same study also reported that most (94.1%) of the women felt worried thinking about a future pregnancy, a concern that was expressed only by some women in our study. The women also expressed a need for more support and care for themselves during the time their babies were admitted to the NICU. Other studies confirm our results, that women want to take the opportunity and advantages of practicing the ‘skin-to-skin’ care at the NICU, even though it could be perceived as difficult due to their own situation^24^. Women with PE experienced more stress and less social support compare to healthy pregnant women^25^. Similar to findings from Australian and Ethiopian studies, our results show that there was a need for additional support during the hospital stay^12,25^. Some women cried during the interview, which should be taken as a warning sign. Particularly, women with severe PE might need help in processing the trauma, suggesting that stress management should be offered routinely^26^. Our results also confirm that there are certain individual needs, especially during the time the women are separated from their newborn. A more optimal organization of care where no separation occurs between parents and newborn would have been preferable. In fact, this is in line with recommendations from WHO. Our results indicate a need for more individual woman-centered care, which also has been described as important in other studies^9,27^. In complicated cases, a teamwork comprising professionals such as obstetricians, pediatricians, NICU nurses, psychologists and midwives should be aimed at so that maternal medical, psychological and care needs are addressed, including information and follow-up of the woman’s partner who may have been extremely distressed in case of a life-threatening situation. Other studies have shown benefits of working in obstetrical teams^28^. Further research may show how we could organize care in a different way to reduce the gaps revealed in our study.

It has been shown that PE is an unknown disease to many women until they actually developed it^13,14^. The knowledge that PE could have long-term consequences with increased risk of cardiovascular disease, diabetes, and stroke^1,7,22^, is often an unknown fact^28^, reported by women in our study. The care of women with PE is unfortunately full of gaps that need attention to become more individualized. About 3% women in Sweden develop PE compared to 1.7% who develop diabetes^29^. Prevention and screening for women, who are at increased risk for gestational diabetes, is performed successfully in Sweden, both during pregnancy and postpartum^30^. The midwives routinely inform the women about the importance of a follow-up visit and their general risks. Similar guidelines and routines might be successful to improve the care for women at risk and/or for women who develop PE. Considering the increased risk of cardiovascular diseases, specific postpartum counselling could be introduced as a routine to give written and oral information according to the new guidelines, which might help to improve long-term outcomes^31^. It is necessary for women diagnosed with PE to get appropriate information about maintaining a healthy lifestyle in order to reduce future risks of the disease. To date, there is neither systematic follow-up to evaluate these risks nor are the women given appropriate information about the additional risks and lifestyle interventions.

Further research needs to investigate if written and oral information about PE, during admission to the hospital and repeated on several occasions, may help the women to understand and cope with their situation better. There is also a need for extra screening regarding their mental health and signs of depression after a pregnancy complicated by severe PE. Our results suggest that midwives and obstetricians must pay more attention to the emotional stress of the women, their need for more personalized and detailed information, as well as providing a detailed plan for follow-up visits postpartum.

### Strengths and limitations

The content analysis was chosen to describe variations regarding similarities and differences in the reported experiences of women regarding the PE information they received. Content analysis is well established and clearly describes the analysis of data^15,18^. The inclusion to this study was at one university hospital with two large clinics at different municipalities. The results derived from the women’s experiences may be transferrable to similar groups but cannot be generalized. The inclusion is limited to only Swedish speaking women. Particularly women from Africa could add knowledge because they have increased risk for severe obstetric complications, and non-Swedish speaking women may have reported a different experience, since they also have a language barrier to handle. Another limitation is that only one multiparous woman was included, which can be explained by the fact that PE is more prevalent in primiparas women.

Throughout the analysis process, several steps were taken to ensure reliability, credibility and trustworthiness^15,18^. Data saturation was reached after 13 interviews with no new topics arising, but two more women were included to confirm saturation. A strength of the study is that all researchers have interprofessional, clinical competence of this care. All the researchers were involved throughout the analysis process and discussions were conducted on how well the themes and sub-themes covered the data. Citations from the original text were selected to illustrate themes and sub-themes, to help the readers evaluate the credibility of the analysis process.

## CONCLUSIONS

The women experienced fragmented obstetrical care and information, when diagnosed with PE. Our findings indicate a need for additional support due to increased stress, worry and despair of being separated from the newborn. Future research investigating specific care-planning and postpartum follow-up visits is suggested as a step to improve care for women with a pregnancy complicated by PE.

## Data Availability

The data supporting this research are available from the authors on reasonable request.

## References

[1] Steegers EA, von Dadelszen P, Duvekot JJ, Pijnenborg R (2010). Pre-eclampsia. Lancet.

[2] Arbets- och Referensgruppen för Perinatologi (2014). Preeklampsi. Report in Swedish.

[3] Graviditetsregistrets Årsrapport 2019 (2019). The Swedish Pregnancy Register - Annual Report 2019. Report in Swedish.

[4] Tranquilli AL, Dekker G, Magee L (2014). The classification, diagnosis and management of the hypertensive disorders of pregnancy: A revised statement from the ISSHP. Pregnancy Hypertens.

[5] National Institute for Health and Care Excellence (2013). Hypertension in pregnancy.

[6] Roberts L, Davis GK, Homer CSE (2019). Depression, Anxiety, and Post-traumatic Stress Disorder Following a Hypertensive Disorder of Pregnancy: A Narrative Literature Review. Front Cardiovasc Med.

[7] Tan MY, Wright D, Syngelaki A (2018). Comparison of diagnostic accuracy of early screening for pre-eclampsia by NICE guidelines and a method combining maternal factors and biomarkers: results of SPREE. Ultrasound Obstet Gynecol.

[8] The Swedish Association of Midwives (2018). Description of Required Competences for Registered Midwives.

[9] Berg M, Asta Ólafsdóttir O, Lundgren I (2012). A midwifery model of woman-centred childbirth care--in Swedish and Icelandic settings. Sex Reprod Healthc.

[10] Bengtsson K Havandeskapsförgiftning. [Preeclampsia]. 1177.se.

[11] Furuta M, Sandall J, Bick D (2014). Women's perceptions and experiences of severe maternal morbidity-a synthesis of qualitative studies using a meta-ethnographic approach. Midwifery.

[12] Roberts LM, Davis GK, Homer CS (2017). Pregnancy with gestational hypertension or preeclampsia: A qualitative exploration of women's experiences. Midwifery.

[13] Carter W, Bick D, Mackintosh N, Sandall J (2017). A narrative synthesis of factors that affect women speaking up about early warning signs and symptoms of pre-eclampsia and responses of healthcare staff. BMC Pregnancy Childbirth.

[14] East C, Conway K, Pollock W, Frawley N, Brennecke S (2011). Women's experiences of preeclampsia: Australian action on preeclampsia survey of women and their confidants. J Pregnancy.

[15] Graneheim UH, Lindgren BM, Lundman B (2017). Methodological challenges in qualitative content analysis: A discussion paper. Nurse Educ Today.

[16] Polit DF, Beck CT (2017). Nursing research: generating and assessing evidence for nursing practice.

[17] Vasileiou K, Barnett J, Thorpe S, Young T (2018). Characterising and justifying sample size sufficiency in interview-based studies: systematic analysis of qualitative health research over a 15-year period. BMC Med Res Methodol.

[18] Graneheim UH, Lundman B (2004). Qualitative content analysis in nursing research: concepts, procedures and measures to achieve trustworthiness. Nurse Educ Today.

[19] Værland IE, Vevatne K, Brinchmann BS (2016). An Integrative Review of Mothers' Experiences of Preeclampsia. J Obstet Gynecol Neonatal Nurs.

[20] Sandall J, Soltani H, Gates S, Shennan A, Devane D (2016). Midwife-led continuity models versus other models of care for childbearing women. Cochrane Database Syst Rev.

[21] Aukes AM, De Groot JC, Wiegman MJ, Aarnoudse JG, Sanwikarja GS, Zeeman GG (2012). Long-term cerebral imaging after pre-eclampsia. BJOG.

[22] Miller EC (2019). Preeclampsia and Cerebrovascular Disease. Hypertension.

[23] Delahaije DH, Dirksen CD, Peeters LL, Smits LJ (2013). Anxiety and depression following preeclampsia or hemolysis, elevated liver enzymes, and low platelets syndrome. A systematic review. Acta Obstet Gynecol Scand.

[24] Blomqvist YT, Nyqvist KH (2011). Swedish mothers' experience of continuous Kangaroo Mother Care. J Clin Nurs.

[25] Sarmasti N, Ayoubi SH, Mahmoudi G, Heydarpour S (2019). Comparing Perceived Social Support and Perceived Stress in Healthy Pregnant Women and Pregnant Women with Preeclampsia. Ethiop J Health Sci.

[26] Sandsæter HL, Horn J, Rich-Edwards JW, Haugdahl HS (2019). Preeclampsia, gestational diabetes and later risk of cardiovascular disease: Women's experiences and motivation for lifestyle changes explored in focus group interviews. BMC Pregnancy Childbirth.

[27] Marsland H, Meza G, de Wildt G, Jones L (2019). A qualitative exploration of women's experiences of antenatal and intrapartum care: The need for a woman-centred approach in the Peruvian Amazon. PLoS One.

[28] Roth H, LeMarquand G, Henry A, Homer C (2019). Assessing Knowledge Gaps of Women and Healthcare Providers Concerning Cardiovascular Risk After Hypertensive Disorders of Pregnancy-A Scoping Review. Front Cardiovasc Med.

[29] Ostlund I, Hanson U (2003). Occurrence of gestational diabetes mellitus and the value of different screening indicators for the oral glucose tolerance test. Acta Obstet Gynecol Scand.

[30] (2008). Mödrahälsovård, Sexuell och Reproduktiv Hälsa. Report in Swedish.

[31] Stefan Hansson S, Hellgren M, Hjertberg R (2019). Riktlinjer för hypertonisjukdomar under graviditet, SFOG 2019-10-23.

